# Structure and Properties of Polylactide Composites with TiO_2_–Lignin Hybrid Fillers

**DOI:** 10.3390/ijms25084398

**Published:** 2024-04-16

**Authors:** Aleksandra Grząbka-Zasadzińska, Agata Piątek, Łukasz Klapiszewski, Sławomir Borysiak

**Affiliations:** Institute of Chemical Technology and Engineering, Faculty of Chemical Technology, Poznan University of Technology, Berdychowo 4, 60-965 Poznan, Poland; agata.piatek3@gmail.com (A.P.); slawomir.borysiak@put.poznan.pl (S.B.)

**Keywords:** biocomposites, polylactide, hybrid materials, TiO_2_, lignin

## Abstract

The research presented in this article focuses on the use of inorganic–organic material, based on titanium dioxide and lignin, as a filler for polylactide (PLA) biocomposites. To date, no research has been conducted to understand the impact of hybrid fillers consisting of TiO_2_ and lignin on the supermolecular structure and crystallization abilities of polylactide. Polymer composites containing 1, 3 or 5 wt.% of hybrid filler or TiO_2_ were assessed in terms of their structure, morphology, and thermal properties. Mechanical properties, including tensile testing, bending, impact strength, and hardness, were discussed. The hybrid filler is characterized by a very good electrokinetic stability at pH greater than 3–4. The addition of all fillers led to a small decrease in the glass transition temperature but, most importantly, the addition of 1% of the hybrid filler to the PLA matrix increased the degree of crystallinity of the material by up to 20%. Microscopic studies revealed differences in the crystallization behavior and nucleation ability of fillers. The use of hybrid filler resulted in higher nucleation density and shorter induction time than in unfilled PLA or PLA with only TiO_2_. The introduction of small amounts of hybrid filler also affected the mechanical properties of the composites, causing an increase in bending strength and hardness. This information may be useful from a technological process standpoint and may also help to increase the range of applicability of biobased materials.

## 1. Introduction

Currently, the search for innovative polymer materials with better functional and ecological properties is becoming more and more common. The circular economy aspect is of great importance to both producers and consumers in many industries. A constantly developing society increasingly pays attention to sustainable development and cares for the environment, resulting in the development of good shopping habits and increased ecological awareness. Consumption causes demand for materials, not only for packaging purposes, that are characterized by increased biodegradability and, in addition, will constitute an attractive product for the consumer in terms of functionality and quality.

Among the biobased polymers, polylactide seems to be the most interesting and commercially used material. Its composites have properties comparable to those of synthetic polymer composites, but they can be advertised as a corn-based, ecofriendly materials. However, the main issue with such materials is the crystallization behavior of PLA. The fact that PLA has a relatively low degree of crystallinity and crystallizes rather slowly is still a limiting agent for its wider use. Therefore, it seems that a solution to overcome issues with the crystallization of PLA is likely to play a vital role in the development of future PLA-based materials. There are different approaches to overcome this problematic issue, and one of them is the formation of composites. To maintain biodegradable properties of the polymer matrix, the fillers used should be “green” as well. Of the different types of fillers, inorganic–organic hybrid materials, combining the advantages of two types of substances, are of special interest.

Lignin is gaining attention as a renewable resource for various applications due to the depletion of conventional fuel reserves. Also, the fact that lignin is a major by-product of a number of industries involved in polysaccharide components from plants for industrial applications makes it an ideal candidate for use in novel materials. Bioconversion of lignin with enzymes [1], conversion with the use of acidic deep eutectic solvents [2], oxidation, demethylation, and demethoxylation [3] are only some of the methods that are used for valorization of lignin in chemicals. Another approach is not to convert lignin, but to use it in its native form. There have been attempts to use lignin for photocatalysis, photovoltaics [4], electrocapacitors, electronics [5], civil engineering [6], etc., showcasing its potential in these fields. There is also a growing trend regarding the use of lignin in polymer biocomposites [7]. This material may address environmental concerns and improve sustainability. Various studies have explored the potential of PLA and lignin composites. Makri et al. [8] demonstrated that reactive in situ processing can significantly enhance the properties of these composites, particularly when using nanolignin. Arjhan et al. [9] found that the addition of lignin can reduce the tensile strength of PLA/lignin films, but also increase their elongation. Wang et al. [10] improved the mechanical properties of PLA composites by incorporating lignin-containing cellulose nanofibrils, while also improving their UV protection and thermal stability. At the same time, Esakkimuthu et al. [11] focused on improving the processability and compatibility of lignin with PLA through etherification, resulting in composites with improved mechanical strength, antioxidant activity, and thermal stability. However, in published studies, the main challenges regarding the use of lignin in PLA are poor thermal stability and lack of compatibility between components—polar filler and nonpolar polymer matrix. The issues with blending lignin with polymers result from strong self-interactions occurring in lignin, which is a complex and polar molecule. These interactions are influenced by the extraction technology used for lignin production, and often require chemical modification or the addition of a compatibilizer for improved dispersion in polymers [12,13].

The problems mentioned above related to the use of lignin can be solved by using titanium dioxide (TiO_2_), a mineral filler of natural origin. Incorporation of TiO_2_ into polymer composites offers a wide range of benefits, including UV protection, mechanical reinforcement, opacity, thermal stability, antibacterial properties, barrier performance, and flame retardancy, making it a versatile additive for various industrial applications. Zhu et al. [14] demonstrated that the dispersion of TiO_2_ in the PLA matrix can be influenced by the processing procedure and that the addition of TiO_2_ can improve the barrier properties of the composites. Man et al. [15] further explored the UV photoresponse effects of different TiO_2_ structures in PLA composites, finding that they can improve the anti-UV performance of the material. It was also proven that titanium dioxide is an active nucleant for PLA [16,17,18]. Although the use of TiO_2_ in polylactide composites can offer some undeniable advantages, there are also some potential disadvantages to consider. The most important, from an economical point of view, is the cost of this filler, which in comparison to other fillers is relatively expensive. Its incorporation can increase the overall cost of production. Thus, it seems that mixing it with a low-cost and highly available lignin could be beneficial. This approach has already been found to be successful for PLA composites with silica/lignin hybrid materials [19,20].

Taking into account all the factors mentioned, it is interesting to investigate whether the use of TiO_2_ and lignin hybrid filler can affect the crystallization behavior of PLA, as well as the formation of a supermolecular structure. Up to now, there have been no publications regarding the use in such a specific role of titanium dioxide–lignin hybrid as a filler for PLA. The objective of this study is to investigate how the introduction of TiO_2_–lignin filler into the polylactide matrix affects the thermal, supermolecular, and macroscopic properties of the material.

## 2. Results and Discussion

### 2.1. Characteristics of Fillers

**Figure 1:**
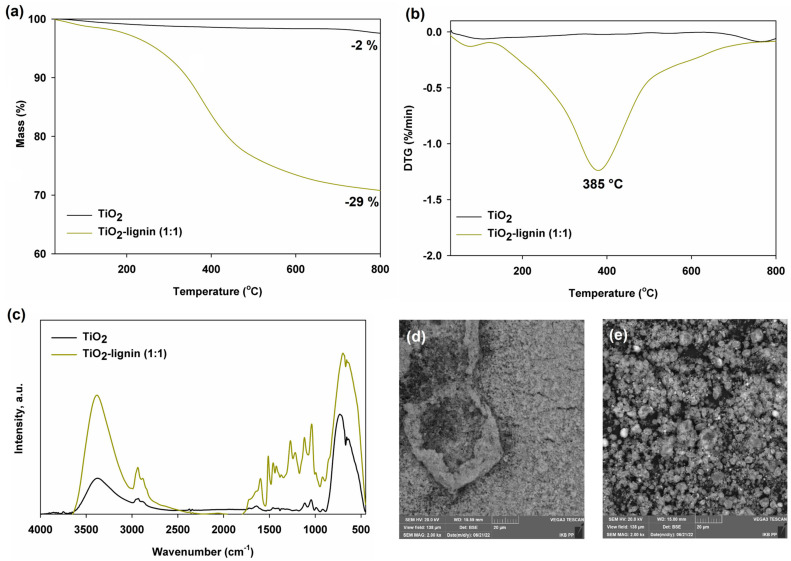
(**a**) TGA curves, (**b**) DTG curves, (**c**) FTIR spectra, and SEM images of starting materials (**d**) TiO_2_, and (**e**) hybrid filler.

TiO_2_–lignin hybrid material is characterized by relatively good thermal stability over the entire range of the analyzed temperature (see Figure 1a,b). 

In the initial phase, at a temperature of about 100 °C, there is a very slight loss of mass, related to the water physically bound to the surface of the system. Subsequently, up to a temperature of approximately 200–220 °C, there is no rapid loss of mass, which could affect the processing of the polymer composite. Only above this temperature does mass loss occur, with its maximum at 385 °C. This is related to the presence of lignin and its progressive thermal decomposition, which has been described in more detail in other publications [21,22]. Undoubtedly, titanium dioxide, which loses only 2% of its initial mass in the analyzed temperature range, improves the thermal stability of the hybrid system.

Figure 1c shows the FTIR spectrum for the TiO_2_–lignin hybrid system, which confirms the effective combination of pure components, leading to the creation of a final class I hybrid material. This is evidenced by slight shifts in the absorption maxima of individual bands in relation to the pure precursors (titanium dioxide and kraft lignin). The spectrum of hybrid material shows characteristic bands assigned to both inorganic components and lignin. Particular attention should be paid to the broad band in the 900–400 cm^−1^ wavenumber range, resulting from the presence of stretching vibrations of the ≡Ti-O-Ti≡ groups in the crystalline structure of titanium dioxide [23]. The broad band in the 3600–3000 cm^−1^ wavenumber range is due to the stretching vibrations associated with the presence of the hydroxyl groups. In turn, the presence of biopolymer in the hybrid material is evidenced by, among others, the band at the maximum wavenumber of 2945 cm^−1^, coming from the stretching vibrations of the C-H groups. Additionally, clearly visible bands in the wavenumber range 1598–1453 cm^−1^ are associated with the presence of an aromatic ring (the aromatic vibrations of the C-C and C=C groups). The presence of lignin in the produced hybrid is also evidenced by the signal from the stretching vibrations of the C-O bonds, visible at the wavenumber of 1258 cm^−1^, as well as the signals at the wavenumbers of 1372 cm^−1^ and 1221 cm^−1^, characteristic of the bending vibrations of the Ar-OH phenolic groups and the stretching vibrations of the C-O(H) and C-O(Ar) groups [19,20,24].

An additional confirmation of the effectiveness of the proposed methodology for obtaining the TiO_2_–lignin hybrid material is the results of the elemental analysis (see Table 1). These results show that, in the case of pure oxide, no elements were observed (except for a small amount of carbon and hydrogen), while in the case of the analyzed hybrid, the presence of elements related to the structure of lignin was observed, including a small amount of sulfur and a significant amount of carbon and hydrogen.

The produced hybrid material is also characterized by relatively good homogeneity, as evidenced by the SEM (scanning electron microscope, image attached in Figure 1e). The micrograph clearly shows individual primary particles that tend to aggregate and consequently agglomerate. This is also confirmed by the results obtained from the Zetasizer Nano analyzer, indicating the presence of particles in the range of 106–1110 nm in the hybrid system. Furthermore, the value of the polydispersity coefficient of 0.686 indicates a relatively good homogeneity of the sample. The tendency of the hybrid system to create larger structures is certainly influenced by the presence of lignin, because the oxide itself, as shown in SEM microphotograph (Figure 1d) and on the basis of the dispersion results presented in Table 1, is characterized by a small particle size distribution and a relatively low polydispersity (PdI) index. Such dependencies can also be found in the available literature on the subject [24,25].

The hybrid material is also characterized by very good electrokinetic stability at pH above 3–4 (see Table 1). Good electrokinetic stability in the case of the produced system is related in particular to lignin and the negative charge on the biopolymer surface, which is characteristic of the functional groups that make up the lignin structure [24,26]. Additionally, a detailed explanation along with the mechanism of action related to the presence of lignin in the hybrid material was presented in detail in our previous study [27]. Pure titanium dioxide in an acidic environment has limited electrokinetic stability, as evidenced by the tendency of this material to reach the isoelectric point at pH around 2 (see Table 1). The detailed mechanism was included in our earlier study [28].

### 2.2. Supermolecular Structure

**Figure 2:**
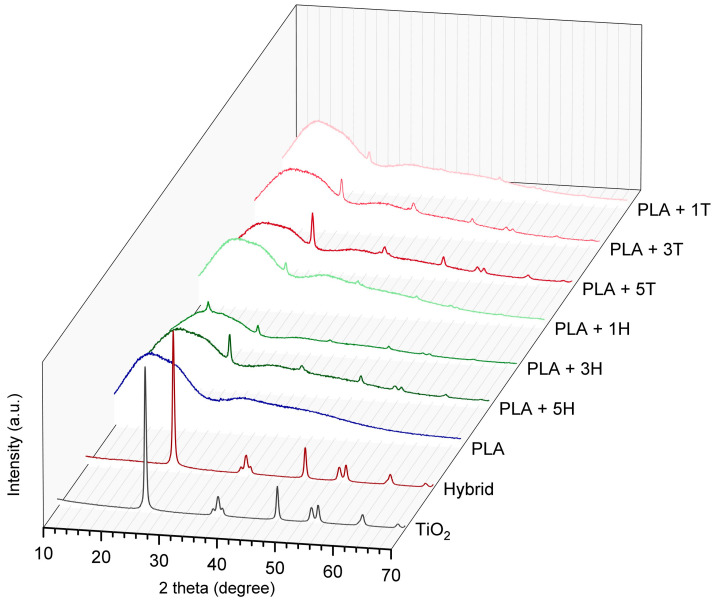
XRD diffractograms for fillers and PLA-based samples.

To determine the supermolecular structure of materials, the X-ray diffraction (XRD) analysis was performed. XRD tests were performed for fillers (titanium dioxide and hybrid), but also for PLA and its composites. The resulting diffractograms are presented in Figure 2.

As can be seen in the figure, diffractograms of TiO_2_ (T) and TiO_2_–lignin hybrid (H) have high-intensity peaks that are the result of XRD on crystallographic planes of TiO_2_. Peaks found at 2θ values of 25.1, 37.7, 47.9, 53.8, 54.9, and 62.4° come from the following crystallographic planes: (101), (004), (200), (105), (211), and (204). According to the literature, they indicate the presence of anatase structure [29] and its JCPDS Card no. is 21-1272. For hybrids, the intensity of these peaks is slightly lower because the amount of anatase in the sample is lower and the lignin itself is an amorphous material, so no XRD maxima can be produced. 

When PLA-based samples are compared, it is visible that all composites have an amorphous region at the 2θ angle of approximately 16° coming from disordered (amorphous) structures of PLA and some small intensity peaks resulting from presence of crystalline anatase. The intensity of these peaks increases as the amount of filler in the samples increases. It is also visible that modification of the system by the addition of amorphous lignin reduces the intensity of the signals; for composites with only TiO_2_, the intensity of maxima is more pronounced. 

For composite PLA + 3H, a sharp peak at 16.9° is observed. This indicates the presence of a crystal structure of PLA. This peak is associated with the formation of ordered crystalline regions in the material [30]. 

### 2.3. Crystallization Studies

**Figure 3:**
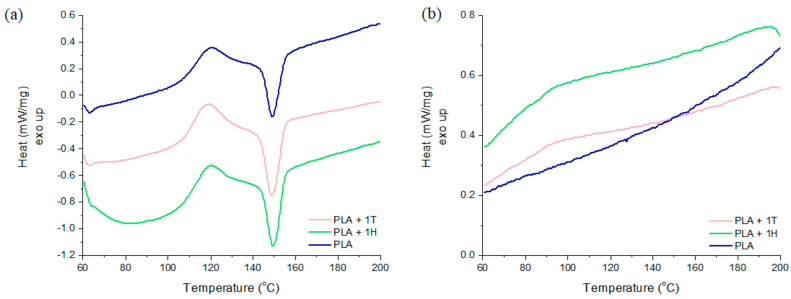
Thermograms of PLA, PLA + 1T, and PLA + 1H recorded during the second run of the DSC: (**a**) heating and (**b**) cooling curves.

Crystallization studies were conducted by means of DSC and PLM methods. First, differential scanning calorimetry was performed. The results are shown in Figure 3, which presents example thermograms, and Table 2 shows summarized data obtained from second runs of DSC.

Analyzing the parameters obtained from DSC measurements, it was found that the addition of the hybrid filler to the PLA matrix resulted in a decrease in the T_g_ value compared to PLA. The values of this parameter were within the range of 61.2–63.3 °C, and for PLA the T_g_ was 63.6 °C. There was also a tendency for the glass transition temperature to drop as the filler content increased. Among all the materials, the composite with 5% TiO_2_ was characterized by the lowest T_g_ value, at 2.4 °C lower than unfilled PLA. This suggests the presence of the plasticizing effect. Therefore, it can be assumed that the fillers used will contribute to increasing the mobility of PLA segments and increasing the flexibility of the obtained material.

The recorded thermograms indicate that unfilled PLA and its composites do not crystallize during cooling. However, the crystallization of all materials (with the exception of PLA with 5% TiO_2_) occurred during heating, in a process called cold crystallization. For unfilled PLA, the exothermic peak of cold crystallization was observed at 120.2 °C. The introduction of filler, regardless of its type and amount, did not significantly affect this parameter, which remained within 119.0–120.6 °C. The fact that the PLA + 5T sample did not exhibit a peak resulting from cold crystallization may suggest that this amount of titanium dioxide inhibits crystallization. Published data indicate that fillers can play a dual role during the cold crystallization process, i.e., act as a crystallization nucleating agent as well as constitute a physical obstacle delaying crystallization [31]. 

When considering melting temperatures, no important differences were observed. The melting temperature for the PLA sample was 149.3 °C, while for the composites it was in the range of 148.1–150.9 °C, which is comparable to published data [32]. 

The unfilled PLA is almost amorphous, and *X_c_* was 1%. The addition of 1%, 3% and 5% TiO_2_ gradually increased the degree of crystallinity from 2% to 7% and finally 8%, which is still rather low. On the other hand, adding 1% of the hybrid filler to the PLA matrix had a pronounced, positive effect on increasing the degree of crystallinity of the material, reaching a value of 20%. Interestingly, composites containing 3% and 5% of hybrid filler were characterized by very low and comparable degree of crystallinity, at approximately 1–2%. 

Similar relationships were reported by Schlarb et al. [33], who also observed that after reaching the limit filler content (in this case SiO_2_) in the PLA matrix, crystallization was limited due to the formation of agglomerates. In this study, the size of the filler could have been the aspect that limited the ability of composites to crystallize. The hybrid filler particles were almost twice as large as those of TiO_2_ (106–1110 nm vs. 91–531 nm) and exhibited some agglomeration. It is likely that a large number of large particles acted as a physical obstacle, making it more difficult for polymeric chains to move.

Moreover, the nucleation effect and the mobility of polymer chains are two processes occurring during the crystallization process. Introducing a relatively large amount of hybrid filler into a PLA matrix is likely to enhance the initial stage of crystallization, which involves nucleus formation, while simultaneously reducing chain mobility.

**Figure 4:**
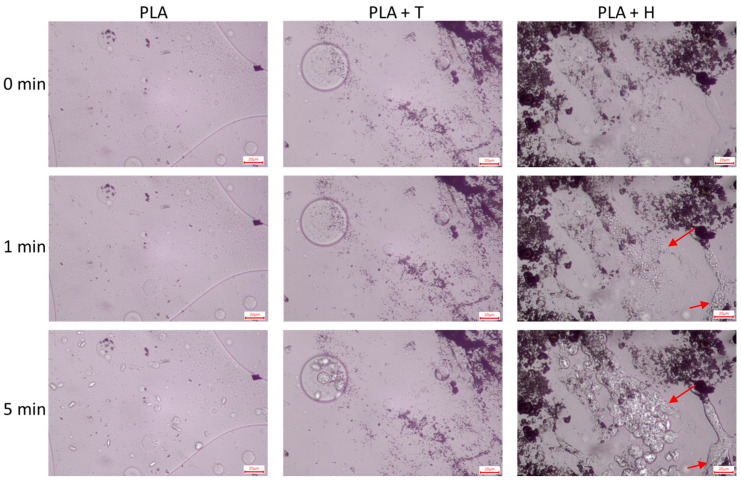
PLM images of PLA and PLA composites taken at the beginning, after 1 min, and 5 min of isothermal crystallization. The scale bar is 20 µm. Arrows show regions with high spherulite density.

The DSC method was applied to assess the nonisothermal crystallization behavior of produced materials, while PLM observations were performed at a constant temperature. Figure 4 presents PLM photographs of samples taken during an isothermal crystallization. 

The microscopic photographs presented show that, in the case of unfilled PLA, spherulites were formed in the mass. Interestingly, when the morphology of the PLA + T and PLA + H systems is analyzed, it is noticeable that the spherulites are also formed on the surface of the filler. This is related to the formation of the so-called transcrystalline layer (TCL) [34]. As proved by Haubruge et al. [35], talc nucleates PET crystallization via an epitaxial mechanism. In the case of PLA, it has been shown that the crystallization half-time can be reduced, even after the addition of 1% talc [36]. Therefore, it can be assumed that it is titanium dioxide that contributes to the formation of TCL. This is in line with our previous study that proved that, in PLA and PLA/lignin samples, only spherulites were visible, while in material with silica–lignin filler, TCL was formed [19].

In the case of composites with hybrid filler, the induction time, that is, the time required to start the formation of spherulites, was lower than for PLA or PLA with TiO_2_. It can also be seen that, for PLA + H composite, small spherulites were clearly visible, whereas in other samples they were not noticeable yet. Furthermore, the PLA + H sample was characterized by the highest density of spherulites. Moreover, in this sample, spherulites were formed in regions where smaller particles and not agglomerates were present. This supports the thesis that the size of the hybrid filler particles is crucial. It also correlates well with the findings of the DSC analysis, in which the PLA + 1H sample had a clearly higher crystallinity degree than other materials. Another aspect may be that polar interactions are formed by weak physical bonds. Hydrogen bonds may have formed between the carbonyl groups of PLA and the functional groups of lignin [37]. 

### 2.4. Mechanical Properties

Mechanical tests were performed to define how the addition of filler affects the mechanical properties of the materials. The tests included uniaxial stretching and Charpy impact strength testing. The results are summarized in Table 3.

Based on the values described in Table 3, it can be concluded that the addition of fillers, regardless of type, had minimal impact on Young’s modulus (YM). The results remain comparable when considering the standard deviation. A similar situation is observed for the impact strength. In terms of tensile strength, the highest value, 65.6 MPa, was reached for unfilled PLA. For composites, the σM parameter was in the range of 63.1–57.9 MPa. Samples filled with titanium dioxide had slightly lower values than those with hybrid filler. However, in both cases, the same tendency was observed: as the amount of filler in PLA matrix increased, the value of the parameter dropped. The elongation at break of the PLA matrix was 6%. When TiO_2_ was added, the parameter changed from 7.8% to 9.1% for PLA + 1T composite. For hybrid-filled samples, the EB values were lower but also dependent on the filler content. The PLA + 1H sample had an EB of 8.1%, while PLA + 5H had only 4.8%. The last parameter tested, the impact strength, for the composites was rather comparable and only slightly lower than for unfilled PLA (2.3–2.6 kJ/m^2^ vs. 2.7 kJ/m^2^).

Interestingly, the tensile strength recorded during the bending test showed some variation. For PLA and its composites with TiO_2_, its value was comparable, at approximately 40 MPa. When hybrid filler was used, σM increased by around 5%, to 42.0–42.7 MPa. For hardness, the situation was different. Composites with TiO_2_ were undoubtedly tougher than unfilled PLA (ca. 50 MPa vs. 46.6 MPa), but there was a small tendency for the parameter to drop as the filler content increased. When hybrid filler was applied, only the composite with 1% filler was tougher (at 48.9 MPa) than PLA itself.

The differences observed for the tensile strength and elongation at break are consistent with the findings of isothermal crystallization studies. The origin of these relationships is believed to be related to both the size and the amount of the filler. Generally, increasing the size of the reinforcement particles leads to a greater debonding of the filler from the polymer matrix [38]. Furthermore, in this study, the presence of lignin was identified as a factor that contributed to a slight decrease in some tensile properties, which has also been reported elsewhere [20,39]. On the other hand, it may be the result of the formation of hydrogen bonds between PLA and lignin, leading to stronger interactions and thus increased stiffness. Although lignin and PLA, due to their ability to form hydrogen bonds, exhibit good compatibility, it has been proved that significant enhancement of mechanical properties often requires fractionation or modification of lignin [40,41,42]. In addition to particle size and bond formation, the macroscopic structure of the material may also play an important role in ensuring appropriate physicochemical properties of PLA-based composites. In general, higher nucleation density results in increased strength and rigidity of polymer composites. This is because the nucleation site acts as the starting point for crystallization, resulting in more uniform and densely packed structures within the polymer matrix and improving the load capacity and deformation resistance. Furthermore, the crystalline regions tend to be harder than the amorphous regions in polymers [43]. Another relevant aspect is that higher nucleation density leads to formation of uniform and finer crystalline structures in polymer matrix. Finer crystallites can contribute to higher hardness by reducing the distance between them, thus creating a more continuous and robust network that resists deformation. Higher nucleation density may increase strength and rigidity, but it may also reduce stiffness and impact resistance. A very high nucleating density can lead to rigid structures that are susceptible to fractures rather than deformations under stress. The balance of nucleation density and other factors, such as resistance, is crucial to prevent excessive brittleness while optimizing resistance.

However, the above results show that the addition of a small amount of hybrid filler to the PLA matrix can help to obtain material with slightly higher elasticity, but at the same time other mechanical properties that are unimpaired, such as tensile strength or Young’s modulus. Low loading of hybrid filler was found to be favorable in terms of enhancement of hardness and bending behavior of materials. 

## 3. Materials and Methods

### 3.1. Materials

Commercial titanium dioxide in the anatase crystallographic form (CAS number: 1317-70-0), supplied by Sigma-Aldrich (Steinheim am Albuch, Germany) and kraft lignin purchased from Merck, Darmstadt, Germany (average Mw ~10,000 g/mol, CAS number: 8068-05-1 were used to produce fillers. Polylactide (PLA) 2003D (Nature Works, MN, USA) was used as a polymer matrix. The content of D-lactide in 2003D PLA is 4.3% [44]. 

### 3.2. Preparation of TiO_2_–Lignin Hybrid

To obtain TiO_2_–lignin hybrid, a method of mechanical grinding of components was used. This method involves mechanical grinding of precursors (titanium dioxide and kraft lignin), with simultaneous mixing using (i) RM100 mortar grinder (Retsch GmbH, Haan, Germany) and then (ii) Pulverisette 6 Classic Line ball mill (Fritsch GmbH, Idar-Oberstein, Germany). The combined application allows for obtaining final material characterized by adequate homogeneity. After milling, the powder was sieved through a sieve with a diameter of 80 µm. For this study, a TiO_2_–lignin hybrid system was prepared with the weight ratio of inorganic to organic parts of 1:1.

### 3.3. Characteristics of Fillers

#### 3.3.1. Fourier Transform Infrared Spectroscopy

To identify the functional groups that were present on the surface of the analyzed materials and to confirm the effective TiO_2_–lignin hybrid material preparation, Fourier transform infrared spectroscopy (FTIR) was used. The samples were analyzed in the form of KBr pellets formed by mixing 1 mg of the sample with 200 mg of anhydrous potassium bromide, over a wavenumber range of 4000–400 cm^−1^, with a resolution of 0.5 cm^−1^ using a Bruker Vertex 70 apparatus (Bruker Optics GmbH & Co. KG, Ettlingen, Germany).

#### 3.3.2. Thermogravimetric Analysis

Jupiter STA 449F3 apparatus (Netzsch, Selb, Germany) was used to perform the thermogravimetric analysis (TGA). Thermal stability tests were carried out under flowing nitrogen (20 cm^3^/min) at a heating rate of 10 °C/min over a temperature range of 25–800 °C, with an initial sample weight of approximately 10 mg.

#### 3.3.3. Dispersive and Morphological Properties

The dispersive and morphological properties of TiO_2_–lignin hybrid and pristine titanium dioxide were determined by particle size measurements using a Zetasizer Nano ZS (0.6–6000 nm) instrument (Malvern Instruments Ltd., Malvern, UK) operating based on the non-invasive backscattering (NIBS) technique. To obtain information on dispersion, particle morphology, and type of agglomeration in the samples, images from a VEGA 3 scanning electron microscope (Tescan Orsay Holding a.s., Brno, Czech Republic) were used.

#### 3.3.4. Elemental Analysis

The elemental content of N, C, H, and S of the powders was assessed using a Vario EL Cube instrument (Elementar Analysensysteme GmbH, Langenselbold, Germany). The samples were placed in the device and combusted in an oxygen atmosphere. After being passed through appropriate materials in a helium stream, the resulting gases were separated in an absorption column.

#### 3.3.5. Electrophoretic Mobility

Electrophoretic mobility was measured at a constant ionic strength of 0.001 M NaCl, and the value of the zeta potential was calculated based on the Henry equation. Titration was performed with a 0.2 M solution of hydrochloric acid or sodium hydroxide. Measurements were made in a pH range from 2 to 10. The mean measurement error of the zeta potential was ±2 mV, and the measurement error of the pH value was ±0.1.

### 3.4. Preparation of Composites

Before processing, the materials were dried in a convection dryer at 60 °C for 8 h. The composites were obtained using a two-step process that included extrusion and injection molding. First, a corotating twin screw extruder (Zamak Mercator, Skawina, Poland) was used with a screw diameter equal to 20 mm and the L/D ratio of 40. The speed of the extruder screws was 8–15 rpm, while the temperatures of the zones were in the range of 150–168 °C. The filler was dosed into the dosing zone using an external hopper at a speed of 8 rpm. The composites were extruded in the form of a circular extrudate and then cut to obtain granules.

The second processing step was injection molding using a Battenfeld UNILOG B2 machine (Formplast GmbH, Mülheim an der Ruhr, Germany). The clamping force of the machine was 500 kN and the diameter of the screw was 30 mm. The temperatures of the injection molding machine zones were set in the range of 160–180 °C. The injection pressure was 1000 bar and then 500 bar. The cooling time was 10 s. A double cavity dumbbell-shaped mold with dimensions 120 mm × 10 mm × 4 mm (length × width × thickness) was used. Mold temperature was kept at 30 °C. At least 10 samples of each material were prepared. 

Using this methodology, composites containing 1%, 3%, and 5% of either TiO_2_ (T) or hybrid filler (H) as well as reference sample of PLA were produced. The exact composition of specimens is presented in Table 4.

**Figure 5:**
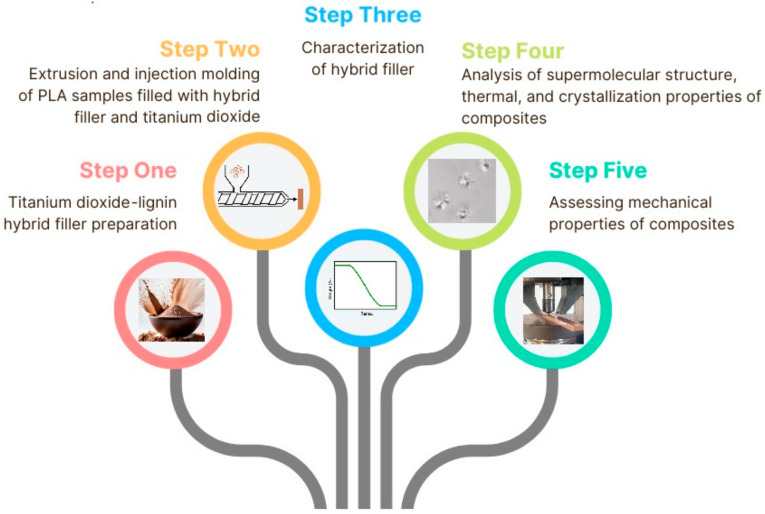
Workflow of the research.

A diagram of the workflow carried out in this study is presented in Figure 5.

### 3.5. Characteristics of Composites

#### 3.5.1. X-ray Diffraction

The supermolecular structure of the materials was analyzed by means of wide angle X-ray scattering (XRD; Rigaku, Tokyo, Japan). CuKα radiation at 40 kV and 30 mA anode excitation was used. The XRD patterns were recorded for 2θ angles from 10 to 70° in steps of 4°/min. The diffraction patterns were used to define the supermolecular structure of the materials of the materials used. The characteristic peaks were assigned to respective crystal planes. 

#### 3.5.2. Differential Scanning Calorimetry

The thermal properties of the samples were evaluated using differential scanning calorimetry (DSC) with a Netzsch DSC 200 instrument (Netzsch, Bavaria, Germany) under a nitrogen atmosphere. Non-isothermal crystallization studies were conducted by initially heating the samples from 40 to 200 °C at a rate of 20 °C/min, followed by a 3 min dwell at this temperature to eliminate any prior thermal or mechanical influences. Subsequently, the samples were cooled to 40 °C at a rate of 5 °C/min and held at this temperature for 1 min. This entire procedure was then repeated. The degree of crystallinity (*X_c_*) of the materials was calculated according to Equation (1).

(1) $$\;X_{c}=\left(\frac{\Delta H_{m}-\Delta H_{cc}}{\Delta H_{m}{}^{\circ}\times \left(1-\frac{\mathrm{wt}.\%\;filler}{100}\right)}\right)\times 100$$

where Δ*H_m_* is the melting enthalpy, Δ*H_cc_* is the enthalpy of the phase produced during cold crystallization, Δ*H_m_*° is the melting enthalpy of a 100% crystalline polylactide (93.0 J/g [26]) and wt.% *filler* is the percentage of filler weight.

In addition, the melting (T_m_), crystallization (T_c_), glass transition (T_g_), and cold crystallization (T_cc_) temperatures were defined using the second run.

#### 3.5.3. Polarized Light Microscopy

The isothermal crystallization of PLA in the presence of TiO_2_ and hybrid fillers was carried out using a Linkam TP93 hot stage optical microscope (Linkam, Redhill, UK) and a Nikon Eclipse polarizing optical microscope, model LV100POL (Nikon, Melville, NY, USA), equipped with a Panasonic CCD GP-KR222 camera (Panasonic, Newark, NJ, USA).

The PLA granules were fragmented into small pieces (approximately 40 μg). A piece was positioned on a microscopic glass slide, onto which a few filler particles were strategically positioned on its surface. The samples were initially heated to 200 °C at a rate of 40 °C/min and held at this temperature for 3 min to eliminate any previous thermal or mechanical effects. After melting, the sample was compressed with another microscopic glass slide. Subsequently, the samples were cooled at 20 °C/min to 142 °C, enabling isothermal crystallization of the PLA. Photographs were taken every 5 s, and analysis was conducted using the ToupView program (ToupTek, Hangzhou, China) to determine the size and growth rate of the PLA spherulites.

#### 3.5.4. Mechanical Properties

PLA-based samples were tested for mechanical properties by means of uniaxial stretching, bending, and Charpy impact strength. A Zwick/Roell Z020 TEW (Ulm, Germany) testing machine was used to perform stretching. Measurements were performed in accordance with the PN-EN ISO 527-1 standard [45]. Dumbbell-shaped samples were stretched at a speed of 50 mm/min and an initial force of 0.2 N. Bending tests were performed using the same machine, in accordance with the PN-EN ISO 178:2019-06 standard [46]. Additionally, the Charpy impact strength of the samples was tested. The measurements were performed in accordance with the PN-EN ISO 179:2023-11 [47], using an Instron CEAST 9050 (Darmstadt, Germany) machine. Samples had a 2 mm V-notch. Brinell hardness tests were performed using an KB150R Brinell tester (KB Prüftechnik GmbH, Hochdorf-Assenheim, Germany), in accordance with the standard PN-EN ISO 2039-1:2004 [48]. During the tests, a ball indenter with a diameter of 5 mm and a test force of 132 N was used. All mechanical tests were performed at a room temperature of 20 °C.

## 4. Conclusions

In this study, for the first time, an attempt was made to understand the impact of hybrid fillers consisting of TiO_2_ and lignin on the supermolecular structure and crystallization abilities of polylactide. FTIR spectroscopy studies led to the conclusion that there is a stable physical interaction between TiO_2_ and lignin, indicating that the creation of a final class I hybrid material was successful. Moreover, analysis confirmed that the hybrid systems exhibit good homogeneity, with a minimal tendency to form agglomerates. Interestingly, the filler that was produced showed very good electrokinetic stability at pH above 3–4. 

As proved by the X-ray diffraction test, the composites prepared with TiO_2_ and TiO_2_–lignin filler were homogeneous. The addition of fillers did not reduce the mechanical properties of the composites. In contrast, the introduction of small amounts of hybrid filler caused an increase in bending strength and hardness. 

Differential scanning calorimetry revealed that the parameter that changed the most was the crystallinity degree—for the composite with 1% hybrid filler it reached the value of 20%. Microscopy studies showed that the use of hybrid filler led to definitely higher nucleation density and reduced induction time, compared to unfilled PLA or PLA with only TiO_2_. 

The observed increase in crystallinity degree is important in terms of widening the possible applications of the produced materials. It is known that polymeric materials with higher *X_c_* have better barrier properties to gasses and moisture, which is an important factor in the packaging industry. Higher degrees of crystallinity are also favorable in terms of the processing of composites, as they allow the process time (i.e., injection molding cycle) to be reduced. The lignin present in the composite may also provide antimicrobial properties to the material.

To conclude, this study correlated the structure of the material, its crystallization behavior, and the nucleation ability of the fillers with the mechanical properties of the composites. Therefore, it provides an improved insight into development of functional, renewably-resourced biocomposites.

## Figures and Tables

**Table 1:** Dispersion properties, elemental analysis and zeta potential vs. pH for titanium dioxide and TiO_2_–lignin (1:1 wt./wt.) hybrid material.

Sample	Dispersion Properties		Elemental Content (%)				pH				
							2	4	6	8	10
	Particle Size Distribution (nm)	Polydispersity Index (PdI)	N	C	H	S	Zeta Potential (mV)				
TiO_2_	91–531	0.198	-	0.1	0.4	-	20.1	−7.2	−20.4	−29.6	−35.1
TiO_2_–lignin (1:1)	106–1110	0.686	0.3	30.1	4.3	1.2	0.1	−29.5	−34.8	−35.9	−40.1

**Table 2:** Summarized data from second runs of DSC.

Sample	T_g_ (°C)	T_cc_ (°C)	T_m_ (°C)	*X_c_* (%)
PLA	63.6	120.2	149.3	1
PLA + 1T	62.6	119.0	149.0	2
PLA + 3T	61.9	120.6	148.1	7
PLA + 5T	61.2	nd	150.9	8
PLA + 1H	63.3	120.2	149.1	20
PLA + 3H	62.0	119.4	148.1	2
PLA + 5H	62.2	119.6	148.6	1

nd—not detected.

**Table 3:** Mechanical properties of PLA-based samples obtained during uniaxial stretching, bending, Charpy impact strength, and hardness tests.

Sample	Uniaxial Stretching			Impact Strength	Bending	Hardness
	YM (MPa)	σM (MPa)	EB (%)	Re (kJ/m^2^)	σM (MPa)	[MPa]
PLA	2290 ± 68	65.6 ± 0.6	6.0 ± 0.9	2.7 ± 0.5	40.2 ± 0.5	46.6 ± 3.1
PLA + 1T	2220 ± 21	62.7 ± 0.5	9.1 ± 0.9	2.5 ± 0.3	40.6 ± 0.4	50.9 ± 1.2
PLA + 3T	2300 ± 12	61.4 ± 0.8	7.8 ± 0.9	2.3 ± 0.2	40.5 ± 0.6	50.1 ± 2.5
PLA + 5T	2222 ± 65	57.9 ± 0.9	8.3 ± 0.7	2.4 ± 0.3	39.4 ± 0.6	49.5 ± 3.3
PLA + 1H	2270 ± 26	63.1 ± 0.3	8.1 ± 0.7	2.3 ± 0.1	42.0 ± 0.1	48.9 ± 1.3
PLA + 3H	2320 ± 27	61.5 ± 0.7	6.6 ± 1.2	2.6 ± 0.4	42.6 ± 0.4	44.1 ± 2.6
PLA + 5H	2360 ± 7	59.3 ± 0.4	4.8 ± 0.7	2.5 ± 0.3	42.7 ± 0.5	45.0 ± 3.0

**Table 4:** List of PLA-based samples.

Polymer Matrix	Filler Type	Filler Content (%)	Sample Name
PLA	-	0	PLA
	TiO_2_	1	PLA + 1T
		3	PLA + 3T
		5	PLA + 5T
	TiO_2_–lignin	1	PLA + 1H
		3	PLA + 3H
		5	PLA + 5H

## Data Availability

Data are contained within the article.

## References

[1] Cajnko M.M., Oblak J., Grilc M., Likozar B. (2021). Enzymatic bioconversion process of lignin: Mechanisms, reactions and kinetics. Bioresour. Technol..

[2] Sosa F.H.B., Bjelić A., Coutinho J.A.P., Costa M.C., Likozar B., Jasiukaitytė-Grojzdek E., Grilc M., da Costa Lopes A.M. (2022). Conversion of Organosolv and Kraft lignins into value-added compounds assisted by an acidic deep eutectic solvent. Sustain. Energy Fuels.

[3] Ročnik T., Likozar B., Jasiukaitytė-Grojzdek E., Grilc M. (2022). Catalytic lignin valorisation by depolymerisation, hydrogenation, demethylation and hydrodeoxygenation: Mechanism, chemical reaction kinetics and transport phenomena. Chem. Eng. J..

[4] Khan A., Nair V., Colmenares J.C., Gläser R. (2018). Lignin-Based Composite Materials for Photocatalysis and Photovoltaics. Top. Curr. Chem..

[5] Wang B., Shi T., Zhang Y., Chen C., Li Q., Fan Y. (2018). Lignin-based highly sensitive flexible pressure sensor for wearable electronics. J. Mater. Chem. C.

[6] Yao H., Wang Y., Liu J., Xu M., Ma P., Ji J., You Z. (2022). Review on Applications of Lignin in Pavement Engineering: A Recent Survey. Front. Mater..

[7] Ma C., Kim T.-H., Liu K., Ma M.-G., Choi S.-E., Si C. (2021). Multifunctional Lignin-Based Composite Materials for Emerging Applications. Front. Bioeng. Biotechnol..

[8] Makri S.P., Xanthopoulou E., Valera M.A., Mangas A., Marra G., Ruiz V., Koltsakidis S., Tzetzis D., Zoikis Karathanasis A., Deligkiozi I. (2023). Poly(Lactic Acid) Composites with Lignin and Nanolignin Synthesized by In Situ Reactive Processing. Polymers.

[9] Arjhan A., Wacharawichanant S., Opaprakasit P. (2022). Influence of Lignin Content on Morphology and Properties of Poly (Lactic Acid)/Lignin Composite Films. Key Eng. Mater..

[10] Wang Y., Liu S., Wang Q., Ji X., Yang G., Chen J., Fatehi P. (2021). Strong, ductile and biodegradable polylactic acid/lignin-containing cellulose nanofibril composites with improved thermal and barrier properties. Ind. Crops Prod..

[11] Esakkimuthu E.S., DeVallance D., Pylypchuk I., Moreno A., Sipponen M.H. (2022). Multifunctional lignin-poly (lactic acid) biocomposites for packaging applications. Front. Bioeng. Biotechnol..

[12] Kun D., Pukánszky B. (2017). Polymer/lignin blends: Interactions, properties, applications. Eur. Polym. J..

[13] Yang S., Yuan T.-Q., Shi Q., Sun R.-C., Han B., Wu T. (2019). Application of Lignin in Thermoplastic Materials. Green Chemistry and Chemical Engineering.

[14] Zhu Y., Buonocore G., Lavorgna M. (2012). Photocatalytic activity of PLA/TiO_2_ nanocomposites and TiO_2_-active multilayered hybrid coatings. Ital. J. Food Sci..

[15] Man C., Zhang C., Liu Y., Wang W., Ren W., Jiang L., Reisdorffer F., Nguyen T.P., Dan Y. (2012). Poly (lactic acid)/titanium dioxide composites: Preparation and performance under ultraviolet irradiation. Polym. Degrad. Stab..

[16] Nomai J., Suksut B., Schlarb A.K. (2015). Crystallization behavior of poly (lactic acid)/titanium dioxide nanocomposites. KMUTNB Int. J. Appl. Sci. Technol..

[17] Song T., Liu M., Tian J., Wang S., Li Q. (2023). Effect of PLA/TiO2/Lg filler competition and synergy on crystallization behavior, mechanics and functionality of composite foaming materials. Polymer.

[18] Deghiche A., Haddaoui N., Zerriouh A., Fenni S.E., Cavallo D., Erto A., Benguerba Y. (2021). Effect of the stearic acid-modified TiO2 on PLA nanocomposites: Morphological and thermal properties at the microscopic scale. J. Environ. Chem. Eng..

[19] Grząbka-Zasadzińska A., Klapiszewski Ł., Bula K., Jesionowski T., Borysiak S. (2016). Supermolecular structure and nucleation ability of polylactide-based composites with silica/lignin hybrid fillers. J. Therm. Anal. Calorim..

[20] Grząbka-Zasadzińska A., Klapiszewski Ł., Borysiak S., Jesionowski T. (2018). Thermal and Mechanical Properties of Silica–Lignin/Polylactide Composites Subjected to Biodegradation. Materials.

[21] Brebu M., Vasile C. (2010). Thermal degradation of lignin—A review. Cellul. Chem. Technol..

[22] Liu Q., Wang S., Zheng Y., Luo Z., Cen K. (2008). Mechanism study of wood lignin pyrolysis by using TG–FTIR analysis. J. Anal. Appl. Pyrolysis.

[23] Erdem B., Hunsicker R.A., Simmons G.W., Sudol E.D., Dimonie V.L., El-Aasser M.S. (2001). XPS and FTIR Surface Characterization of TiO_2_ Particles Used in Polymer Encapsulation. Langmuir.

[24] Klapiszewska I., Parus A., Ławniczak Ł., Jesionowski T., Klapiszewski Ł., Ślosarczyk A. (2021). Production of antibacterial cement composites containing ZnO/lignin and ZnO–SiO2/lignin hybrid admixtures. Cem. Concr. Compos..

[25] Klapiszewski Ł., Grząbka-Zasadzińska A., Borysiak S., Jesionowski T. (2019). Preparation and characterization of polypropylene composites reinforced by functional ZnO/lignin hybrid materials. Polym. Test..

[26] Wysokowski M., Klapiszewski Ł., Moszyński D., Bartczak P., Szatkowski T., Majchrzak I., Siwińska-Stefańska K., Bazhenov V.V., Jesionowski T. (2014). Modification of chitin with kraft lignin and development of new biosorbents for removal of cadmium (II) and nickel (II) ions. Mar. Drugs.

[27] Klapiszewski Ł., Nowacka M., Milczarek G., Jesionowski T. (2013). Physicochemical and electrokinetic properties of silica/lignin biocomposites. Carbohydr. Polym..

[28] Jędrzejczak P., Parus A., Balicki S., Kornaus K., Janczarek M., Wilk K.A., Jesionowski T., Ślosarczyk A., Klapiszewski Ł. (2023). The influence of various forms of titanium dioxide on the performance of resultant cement composites with photocatalytic and antibacterial functions. Mater. Res. Bull..

[29] Reyes-Coronado D., Rodríguez-Gattorno G., Espinosa-Pesqueira M., Cab C., De Coss R.d., Oskam G. (2008). Phase-pure TiO2 nanoparticles: Anatase, brookite and rutile. Nanotechnology.

[30] dos Santos F.A., Valle Iulianelli G.C., Bruno Tavares M.I. (2017). Development and properties evaluation of bio-based PLA/PLGA blend films reinforced with microcrystalline cellulose and organophilic silica. Polym. Eng. Sci..

[31] Li H., Huneault M.A. (2007). Effect of nucleation and plasticization on the crystallization of poly (lactic acid). Polymer.

[32] Kaseem M., Hamad K., Ur Rehman Z. (2019). Review of Recent Advances in Polylactic Acid/TiO_2_ Composites. Materials.

[33] Saiprasit P., Schlarb A. (2021). The effect of the compounding procedure on the morphology and mechanical properties of PLA/PBAT-based nanocomposites. Int. Polym. Process..

[34] Looijmans S.F.S.P., Spanjaards M.M.A., Puskar L., Cavallo D., Anderson P.D., van Breemen L.C.A. (2022). Synergy of Fiber Surface Chemistry and Flow: Multi-Phase Transcrystallization in Fiber-Reinforced Thermoplastics. Polymers.

[35] Haubruge H.G., Daussin R., Jonas A.M., Legras R., Wittmann J.C., Lotz B. (2003). Epitaxial Nucleation of Poly(ethylene terephthalate) by Talc:  Structure at the Lattice and Lamellar Scales. Macromolecules.

[36] Ke T., Sun X. (2003). Melting behavior and crystallization kinetics of starch and poly(lactic acid) composites. J. Appl. Polym. Sci..

[37] Spiridon I., Tanase C.E. (2018). Design, characterization and preliminary biological evaluation of new lignin-PLA biocomposites. Int. J. Biol. Macromol..

[38] Omar M.F., Akil H.M., Ahmad Z.A. (2013). Particle size—Dependent on the static and dynamic compression properties of polypropylene/silica composites. Mater. Des..

[39] Li J., He Y., Inoue Y. (2003). Thermal and mechanical properties of biodegradable blends of poly(L-lactic acid) and lignin. Polym. Int..

[40] Shakoor Shar A., Wang N., Chen T., Zhao X., Weng Y. (2023). Development of PLA/Lignin Bio-Composites Compatibilized by Ethylene Glycol Diglycidyl Ether and Poly (ethylene glycol) Diglycidyl Ether. Polymers.

[41] Yang W., Dominici F., Fortunati E., Kenny J.M., Puglia D. (2015). Effect of lignin nanoparticles and masterbatch procedures on the final properties of glycidyl methacrylate-g-poly (lactic acid) films before and after accelerated UV weathering. Ind. Crops Prod..

[42] Zhu J., Xue L., Wei W., Mu C., Jiang M., Zhou Z. (2015). Modification of lignin with silane coupling agent to improve the interface of poly (L-lactic) acid/lignin composites. BioResources.

[43] Flores A., Ania F., Baltá-Calleja F.J. (2009). From the glassy state to ordered polymer structures: A microhardness study. Polymer.

[44] Zhou H., Zhao M., Qu Z., Mi J., Wang X., Deng Y. (2018). Thermal and Rheological Properties of Poly(lactic acid)/Low-Density Polyethylene Blends and Their Supercritical CO_2_ Foaming Behavior. J. Polym. Environ..

[45] (2018). Differential Scanning Calorimetry (DSC)—Part 3: Determination of Temperature and Enthalpy of Melting and Crystallization.

[46] (2019). Plastics—Determination of Flexural Properties.

[47] (2023). Plastics—Charpy Impact Testing—Part 1: Non-Instrumental Impact Testing.

[48] (2004). Plastics—Determination of Hardness—Part 1: Ball Indentation Method.

